# Anti-Inflammatory Thalidomide Improves Islet Grafts Survival and Functions in a Xenogenic Environment

**DOI:** 10.1371/journal.pone.0006312

**Published:** 2009-07-20

**Authors:** Chunguang Chen, Carina Kuehn, Reinhard G. Bretzel, Thomas Linn

**Affiliations:** Medical Clinic and Policlinic 3, Justus-Liebig University Giessen, Giessen, Germany; University of Bremen, Germany

## Abstract

Thalidomide possesses both anti-inflammatory and anti-angiogenic properties. This study investigates its potential application in islet transplantation with a xenogenic transplantation model. Transplantation was performed using C57Bl/6 mice and NMRI nu/nu mice as recipients of porcine islets. Moreover, islet graft vasculature and inflammation were investigated to identify the mechanisms of thalidomide action. In the immunocompetent environment of C57Bl/6 mice, a fast graft rejection was observed. The group treated with thalidomide 200 mg/kg BW per day achieved and maintained euglycemia in the complete observation period for 42 days. The treated mice had more functional islet graft mass with less leukocyte infiltration. The pro-inflammatory TNF-α and VEGF content in islet grafted kidneys was significantly lowered by the treatment. By comparison, thalidomide was not effective in improving graft survival in immunocompromised nude mice. It strongly inhibited the VEGF and TNF-α-induced endothelial proliferation of isolated pig islets in a dose dependent manner. The magnitude of thalidomide's inhibitory effect was nearly identical to the effect of VEGF- receptor 2 inhibitor SU416 and anti-TNF-receptor 1 neutralizing antibody, and was reversed by sphingosine-1-phosphate. In conclusion, the anti-inflammatory effect of thalidomide improved islet graft survival and function in a transplantation model with a maximum immune barrier.

## Introduction

Islet transplantation represents a potential cure for type 1 diabetes. Great improvements have been achieved since the introduction of the Edmonton protocol [1], [2]. However, two or more donor pancreata are routinely needed for one recipient, thus representing a major obstacle for the widespread application of islet transplantation [1], [3], [4]. Conversely, it is generally believed that only a small fraction of the transplanted islets successfully engraft [5], indicating that more effort is needed to improve the current protocol. Both non-immune and innate immunological factors have been indicated in the primary non-function and failure of islet graft [6], [7]. In islet xenotransplantation, which could potentially resolve the extreme shortage of human donor pancreata by using the pig as the source of islets, even more severe inflammation and early graft loss were observed [8], [9], [10], [11].

Among the different mechanisms and molecules that are implicated in early graft dysfunction and death, tumor-necrosis factor alpha (TNF-α) is one of the most widely studied [12]. TNF-α is a key regulator of systemic inflammation and a stimulator of the acute phase reaction. Several studies have demonstrated that the plasma level of TNF-α is increased by hyperglycemia and diabetes [13], [14], [15]. In islet transplantation, islet-toxic TNF-α has been shown to be produced locally at the graft site by recipient macrophages, as well as by resident macrophages within the islet itself [16], [17]. Inhibition of TNF-α production is shown to improve islet survival and functions [17]. On the other hand, although TNF-α has been validated as a drug target with Remicade and Enbrel available as prescription medications, both are large macromolecules and require injection which limit their clinical applications.

Thalidomide is a TNF-α inhibitor that has anti-angiogenic, immunomodulatory and anti-inflammatory effects [18], [19]. It was first introduced in the 1950s as a sedative and quickly withdrawn in 1961 because of its teratogenic effects. However, it was reintroduced in 1997 as an immunomodulator and is now successfully used in the treatment of multiple myeloma, leprosy and various autoimmune diseases [20]. Thalidomide has potent anti-inflammatory activity. It inhibits the production of TNF-α in monocytes and macrophages by increasing the rate of TNF-α mRNA degradation [21], [22]. In addition, thalidomide is capable of blocking NF-κB activation via a mechanism that involves suppression of IκB kinase activity in T lymphocytes [23]. Furthermore, thalidomide treatment was shown to down-regulate the increased expression of P-selectin and intercellular adhesion molecule (ICAM-1) in injured endothelium, thus reducing neutrophil recruitment [24].

Given the unique immunomodulatory property of thalidomide, this study was designed to evaluate its therapeutic potential on islet graft survival, functions and revascularization using an islet xenotransplantation model. We hypothesized that the anti-inflammatory effect of thalidomide, especially the inhibition of TNF-α production, could protect the islet graft from the destructive post-transplantation inflammatory events and improve the transplantation outcome. We investigated the effects of thalidomide treatment on blood glucose profile, islet graft mass, leukocyte infiltration and TNF-α content in the graft, graft surface blood flow and vascular density in the recipients after porcine islets were transplanted into C57Bl/6 mice. The effect of thalidomide on VEGF and TNF-α-induced angiogenesis was studied using an *ex vivo* pig islet sprout assay. In order to exclude its anti-inflammatory effect, the influence of thalidomide's anti-angiogenic effect on islet xenograft survival and functions was studied using the immunocompromised NMRI nu/nu mice as recipients.

## Materials and Methods

### Pig islet isolation

Pig islets were isolated at the Islet Isolation Facility of Medizinische Klinik und Poliklinik III am Universitätsklinikum Gieβen using previously described techniques of collagenase digestion and Ficoll purification [25], [26]. After isolation, the quality of islet isolation was evaluated by Trypan Blue exclusion, dithizone staining and glucose-stimulated insulin secretion to check viability, purity and function. The purity varied from 95–100% and viability was more than 95% for subsequent experiments. The islets were cultivated in a humidified air atmosphere after isolation until use within 5 days.

Animal research was approved by Regional Commission Giessen, Germany under the code number GI20/11-Nr.15/2006. Animal husbandry was performed according to the German Animal Welfare Law as published in the latest version under http://bundesrecht.juris.de/tierschg.

### Islet transplantation

Before transplantation, diabetes was induced in the recipients by a single injection of 200 mg/kg streptozotocin (Sigma-Aldrich) intravenously into the tail vein, and blood glucose levels were determined by Glucometer Elite (Bayer). Mice with a blood glucose value of more than 300 mg/dL for at least two consecutive days were used as transplant recipients.

Recipients were anaesthetized with 2.5% avertine (200 µL/100 g, Sigma), and the kidney was assessed by an incision of the left flank. 3,000 IEQ of porcine islets were transplanted beneath the kidney capsule of diabetic mice. The wound was closed in two layers with absorbable sutures. Blood-glucose levels were analyzed during the postoperative days.

Thalidomide, at a dose of 200 mg per kg body weight [24], was dissolved in dimethylsulfoxide (DMSO) and a daily intraperitoneal injection was given to mice in the treated groups starting at three days prior to transplantation and continuing through to the end of the experiments. Preliminary experiments indicated that the vehicle, DMSO, was not toxic to the animals.

The kidney, bearing islet grafts, was recovered from the body for histological analysis or protein extraction after 14 days in the C57Bl/6 control group (vehicle only) and one thalidomide-treated C57BL/6 group, and after 42 days in the other groups. Three days after explantation of the graft, the blood-glucose levels were analyzed to verify the recurrence of diabetes.

### Measurements of blood flow in transplanted islets

The blood flow of the islet graft was measured as previously described [27]. Briefly, the blood perfusion of the islet graft and the adjacent renal cortex was measured by laser-Doppler flow detection (PF 4001–2, Perimed, Stockholm, Sweden) with a needle probe (411 mm tip; outside diameter, 0.45 mm). The flow probe was positioned perpendicular to the immobilized tissue surface by the use of a micromanipulator, and care was taken not to cause any compression of the tissue. The blood flow was calculated as the percentage of the graft flow rate to kidney surface flow rate.

### Protein analysis of the islet grafts

For protein extraction the frozen grafts were thawed and lyzed for 30 min by 10% dithiothreitol 10 mM, 1% protease inhibitor cocktail, and 89% buffer AM1 (Active Motif, Rixensart, Belgium). The supernatant was collected after two centrifugation steps at 3,000 g for 10 min. In the resulting lysate protein concentrations were determined using specific mouse immunoassays for insulin (DRG, Marburg, Germany), VEGF, or TNF-α (R&D systems, Abingdon, UK) according to the manufacturers' instructions.

### Myeloperoxidase (MPO) assay

Grafted kidneys were homogenized in 1 ml PBS at 4°C using a Polytron homogenizer (five bursts of 10 s each at maximum speed). 250 µl of the homogenate was added to 250 ml hexadecyltrimethylammonium bromide, vortexed, and incubated for 2 min. After centrifugation, the supernatant was collected and assayed for MPO activity by adding 55 µl tetramethylbenzidine substrate to 30 µl of the supernatant. The absorbance was read at 630 nm at intervals of 30 s for 2 min.

### Immunohistology and Measurement of Vascular Density

Whole graft-containing kidneys were embedded in Paraplast. Each kidney block was serially sectioned (7 µm) throughout its length. Endothelial cells (ECs) and microvessels were identified by staining with biotinylated Bandereira simplicifolia (BS-1, Sigma) as previously described [27], [28]. Briefly, slides were pretreated with neuraminidase type X (Sigma) and incubated with biotinylated BS-1 at 4°C overnight. After three rinses with PBS, they were incubated with StreptABComplex (Dako) for 30 minutes at room temperature. Subsequently, fuchsin and 1 mM levamisole were added and developed. Finally, the sections were counterstained with hematoxylin, cleared and mounted. Vascular area cross sections within grafts were counted for percentage of lectin-positive area in 7 to 10 5000-µm^2^ fields in each section with a light microscope (magnification 200×) combined with an image analysis system (Motic, Wetzlar, Germany). Vascular density was expressed as percentage of vascular area to total area examined.

### Islet Sprouting

Three hundred microliters of bovine fibrinogen dissolved in Dulbecco's PBS at 1.7 mg/ml, containing aprotinin was added to wells of a 24-well plate. After a 24-h pre-culture period, 20–30 islets were embedded into each of the prepared wells. Thalidomide or vehicle (DMSO) was subsequently added prior to fibrin polymerization induced by addition of 5 µl of thrombin (1000 U/ml). Anti-TNFR1 neutralizing antibody (clone 55R-170, R&D systems) was also added before fibrin polymerization. Additionally, the flk-1 (VEGFR 2) inhibitor, SU416, and the enhancer of VEGF-induced angiogenesis, sphingosine-1-phosphate (S1P) (both purchased from Sigma-Aldrich) were used prior to fibrin polymerization. Cultivation of islets was carried out in medium containing bFGF, VEGF or TNF-α (all human recombinant, BD Biosciences, San Jose, USA) for 48 h or time indicated at the concentrations specified. Gels were fixed with 3% formaldehyde and stained with crystal violet. Washed gels were transferred onto slides and fixed with Kayser's glycerin gelatin. Islet vitality was tested prior to the angiogenesis assay using Trypan Blue staining and by determination of islet morphology in the fixed gels. Vital islets did not disperse and had a smooth shape without cellular protrusions. Their angiogenic potential was quantified by determining the percentage of sprouting islets. Islets containing at least one capillary sprout were considered to be angiogenic. At any tested condition at least three independent experiments were performed.

### Statistics

Results are given as mean±SEM. Statistical significance was determined using student's *t*-test or one-way ANOVA with posthoc Bonferroni's test, as appropriate. A value of p<0.05 was considered statistically significant.

## Results

### Death rate in transplanted C57Bl/6 mice reduced by thalidomide

**Figure 1:**
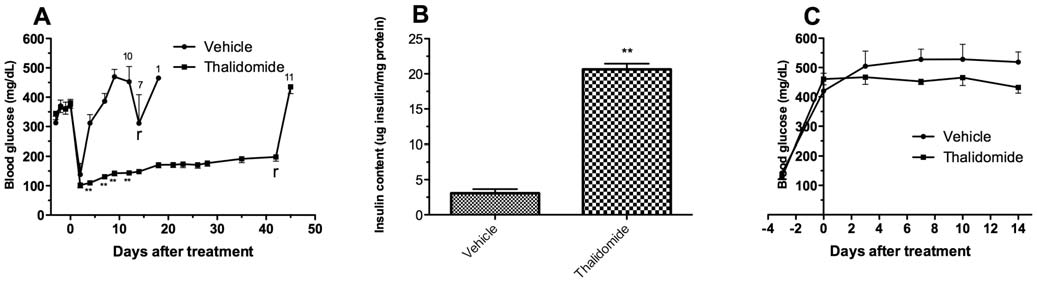
Effect of thalidomide treatment on glycemic control in C57Bl/6 mice. Blood glucose levels of C57Bl/6 mice with islet transplantation (A, n = 11 in vehicle control group and n = 12 in thalidomide treated group) or without islet transplantation (C, n = 6). In islet transplantation groups, nephrectomy of the graft bearing kidney was performed on day 14 and day 42, in control mice or treated mice respectively, followed by blood glucose determination 3 days later. The number of the surviving animals is indicated above the curve. Insulin content in the grafted kidneys is shown in figure B. ^r^removal of graft kidney; ^11^n = 11; ^10^n = 10; ^7^n = 7; ^1^n = 1; ^**^p<0.01 vs control mice.

As shown in Figure 1A where the surviving animal numbers are indicated above the blood glucose curve, the death rate prior to retrieval of the graft-bearing kidney was higher in the C57Bl/6 vehicle group compared to the thalidomide treated C57Bl/6 mice (4/11 vs 0/12). In critical situations, blood glucose values of the surviving mice in the vehicle group dropped down dramatically indicating that the animals were going to die. This is proved to be true by the fact that only 1 mouse from this group survived after removing the grafted kidney while 11 out of 12 mice in the treated group survived.

### Thalidomide improved glycemic control and islet graft survival in C57Bl/6 mice recipients

A rapid rejection was found in the vehicle C57Bl/6 mice group after transplantation with blood glucose values dramatically increased (Figure 1A). All the mice became diabetic again and 36% (4/11) of them died due to severe diabetes before the retrieval of the graft at day 14. In contrast, the thalidomide-treated mice were restored with euglycemia for the period of observation although the blood glucose values increased slowly following transplantation.

Consistently, a significantly higher graft insulin content was found in the thalidomide-treated C57Bl/6 group at day 42 compared to the vehicle group (µg insulin/mg protein: 20.63±0.82 *vs* 3.08±0.57, p<0.01; Figure 1 B), indicating that a greater islet graft mass resulted from the treatment.

After the removal of the islet graft-bearing kidneys, all the mice in both groups became diabetic again with six of seven survived mice died in the vehicle group (Figure 1A), indicating that the islet grafts were taking effect before the retrieval.

In a separate experiment, thalidomide administration without islet transplantation did not have a noticeable impact on severity of hyperglycemia in diabetic mice. As shown is Figure 1C, all the mice in both groups remained diabetic during the observation period.

### Protection from leukocyte infiltration within xenograft by thalidomide

**Figure 2:**
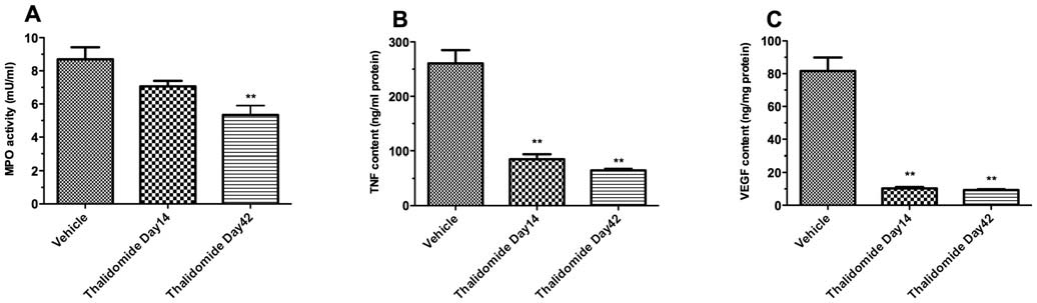
Effects of thalidomide treatment on MPO activity, TNF-α and VEGF content within the grafted kidneys. The retrieved grafted kidneys from C57Bl/6 recipients were subjected to Myeloperoxidase (MPO) assay (A) or specific mouse immunoassay for TNF-α (B) or VEGF (C). N = 6 in each assay. ^**^p<0.01 vs vehicle control mouse kidneys.

Neutrophilic granulocytes have been shown to be the predominant cell type infiltrating islets *in vitro* [29]. Therefore, we investigated the effect of thalidomide treatment on neutrophil infiltration by measuring the MPO activity in islet graft. Tissue MPO activity correlates with the number of neutrophils extravasated into the xenograft [30]. As shown in Figure 2A, we observed decreased neutrophil influx in the kidney of C57Bl/6 mice treated with thalidomide for 42 days (MPO mU/ml: 5.0±0.7 *vs* 8.4±1.2, p<0.01 *vs* vehicle; day 42).

### Reduction of TNF-α and VEGF content in xenograft by thalidomide

Murine TNF-α and VEGF content in the grafted kidneys was evaluated by ELISA. The TNF-α content in the grafted kidneys in both thalidomide treated groups at day14 and day 42 was significantly lower than in the vehicle group (Figure 2B) on the day of graft retrieval, while there are no significant differences between thalidomide groups at day 14 or day 42. Thalidomide treatment also significantly reduced VEGF content in these kidneys (Figure 2C).

### Thalidomide inhibited sprout formation in pig islets

**Figure 3:**
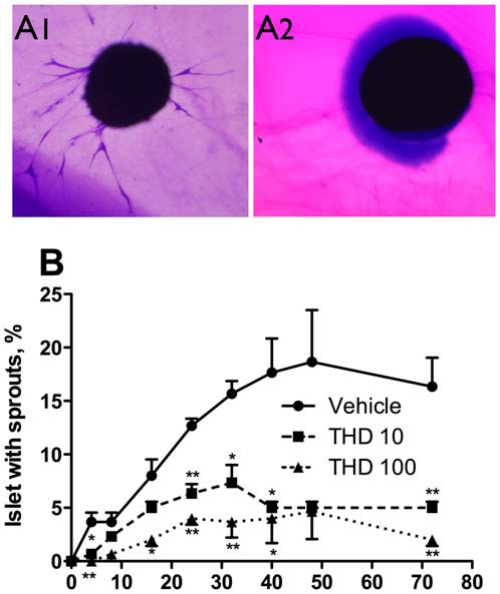
Time curve and dose-dependency of thalidomide's effect on sprout formation of pig islets. After cultivation of pig islets in medium containing 5 ng/ml bFGF and different concentration of thalidomide, the gels were fixed at the indicated times, stained and counted for percentage of islets with sprouts. A: Representative pictures of pig islet in the sprouting assay with or without sprouting activity (A1 and A2, respectively). B: The time curve of islet sprouting within 72 hours after cultivation. N = 3 in each time point. ^Veh^vehicle (DMSO); ^THD 10^thalidomide 10 µg/ml; ^THD100^thalidomide 100 µg/ml. ^*^p<0.05 *vs* Veh; ^**^p<0.01 *vs* Veh.

The effect of thalidomide treatment on angiogenesis of pig islets was examined by the *ex vivo* sprout formation assay of islet endothelial cells embedded within a fibrin gel. Representative pictures of pig islet with or without sprouting activity in the assay are shown in Figure 3A (A1 and A2 respectively). The time course and the percentage of sprouting pig islets were studied. As shown in Figure 3B, the tube-like structures protruding from the islets peaked at 48 hours after cultivation. On average, 18.7±4.8% of these islets formed tube-like structures protruding from the islets in the gel in the presence of 5 ng/ml bFGF. When different doses of thalidomide were added to the medium, islet sprout formation was reduced in a dose-dependent fashion. We had previously demonstrated that the tubes consisted of CD–31-positive endothelial cells [31].

**Figure 4:**
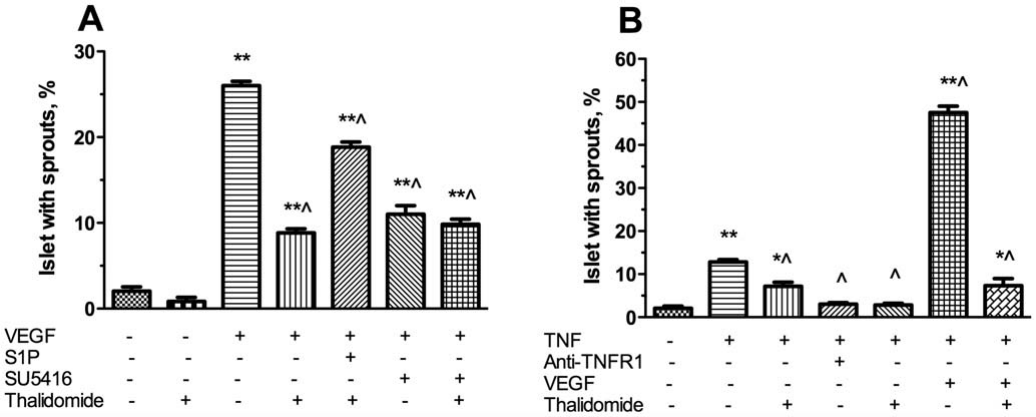
Effect of thalidomide on VEGF or TNF-α induced sprout formation in pig islets. 50 ng/ml VEGF, 0.5 µM S1P, 5 µM SU5146, 50 ng/ml TNF-α, 10 µg/ml Anti-TNFR1, 50 µg/ml thalidomide, used alone or in different combinations was added to the medium in the above mentioned pig islet sprouting model. The percentage of islets with sprouts was counted. N = 6 in each condition. ^*^p<0.05 vs vehicle control; ^**^p<0.01 vs vehicle control; ^ ^^p<0.01 vs 50 ng/ml VEGF (A) or 50 ng/ml TNF-α (B).

To investigate the mechanisms of thalidomide's effects on islet sprouting, we examined the effect of thalidomide on VEGF or TNF-α induced angiogenesis in this model. As shown in Figure 4A, the addition of 50 ng/ml VEGF increased the percentage of pig islets with sprouts from 2±0.52 in the vehicle control to 26±0.52 (p<0.01). The effect was partially inhibited to 8.83±0.48 (p<0.01 *vs* VEGF group) by addition of 50 µg/ml thalidomide. S1P has been reported to antagonize thalidomide's depletion of VEGFR2 and neuropilin in human umbilical vein endothelial cells [32]. We found that S1P increased islet endothelial sprouting in the presence of thalidomide (18.8±0.6, p<0.01 *vs* VEGF+thalidomide). In addition, thalidomide's inhibitory effect was mimicked by the VEGFR2 inhibitor, SU416 (11.0±1.0, p<0.01 *vs* VEGF). Furthermore, the combination of thalidomide and SU416 did not have an accumulative inhibitory effect (9.8±0.6, p<0.01 *vs* VEGF, NS *vs* thalidomide). These results indicate that thalidomide's action in islet endothelial cells was predominantly mediated by VEGFR2.

Similarly, the addition of 50 ng/ml TNF-α increased the percentage of pig islets with sprouts to 12.83±0.54 (p<0.01 *vs* vehicle, Figure 4B). Thalidomide antagonized TNF-α-induced sprouting of islets to 7.17±0.98%(p<0.01 *vs* TNF-α alone). In comparison, an anti-TNFR1 neutralizing antibody [33] was more effective than thalidomide in inhibiting TNF-α induced sprouting (3.0±0.4%, p<0.01 *vs* TNF-α). The combination of thalidomide and anti-TNFR1 did not have an accumulative effect (2.8±0.4%, p<0.01 *vs* TNF-α, NS *vs* thalidomide). The combination of VEGF and TNF-α potentiated the sprouting rate to 47.5±1.5% (p<0.01 *vs* VEGF or TNF-α alone).Thalidomide was capable of inhibiting VEGF/TNF-α induced sprouting significantly to 7.33±1.63 (p<0.01).

### Thalidomide restrained islet graft survival and function in NMRI nu/nu mice

**Figure 5:**
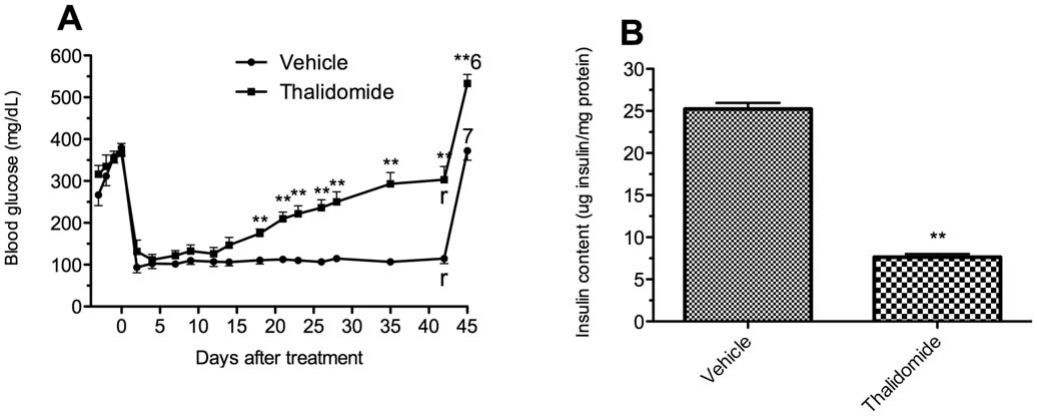
Effect of thalidomide treatment on glycemic control in NMRI nu/nu mice. Blood glucose levels of nu/nu mice (n = 8, except indicated) in vehicle group and thalidomide treated group. Nephrectomy of the graft bearing kidney was performed on day 42, followed by blood glucose determination 3 days later. Insulin content in the grafted kidneys is shown in figure B. ^r^removal of graft kidney; ^7^n = 7;^ 6^n = 6;^ **^p<0.01 vs vehicle control mice.

In order to exclude its anti-inflammatory effect, thalidomide's anti-angiogenic effect on islet grafts was studied *in vivo* by using the immunocompromised NMRI nu/nu mice as recipients of pig islets. As shown in Figure 5A, diabetic nu/nu mice in the vehicle group exhibited stable euglycemia after islet transplantation until the removal of the graft-bearing kidney. In the thalidomide-treated group, euglycemia was achieved but blood glucose values increased continuously from day 12 post-transplantation with recurrence of diabetes in 7 of 8 (87.5%) mice before the recovery of the grafted kidneys.

Consistently, less insulin content was found in thalidomide-treated grafted kidneys compared to those of the vehicle grafted kidneys (µg insulin/mg protein: 7.63±0.38 *vs* 25.23±0.74, p<0.01; Figure 5B).

### Thalidomide impaired graft revascularization in NMRI nu/nu mice

**Figure 6:**
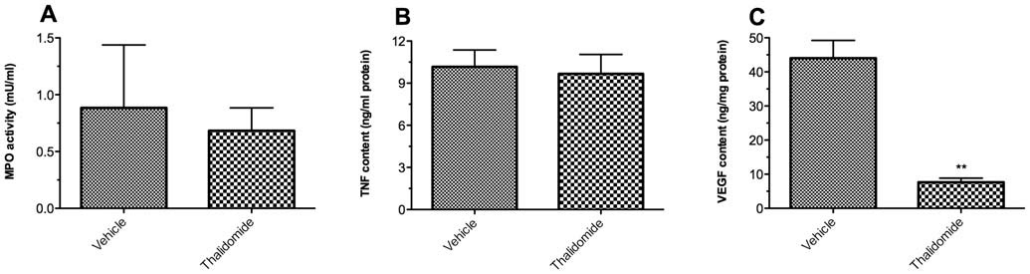
Effects of thalidomide treatment on MPO activity, TNF-α and VEGF content in the grafted kidneys. The retrieved grafted kidneys from NMRI nu/nu recipient mice were subjected to Myeloperoxidase (MPO) assay (A) or specific mouse immunoassay for TNF-α (B) or VEGF (C). N = 6 in each assay. ^**^p<0.01 vs vehicle control mouse kidneys.

As immunocompromised, the nu/nu mice did not mount a cellular immune response of graft rejection that was confirmed by MPO assay and graft TNF-α ELISA. As shown in Figure 6, the MPO activity in the grafted kidneys of nu/nu mice recipients was very low (0.9±0.6 mU/ml). Thalidomide treatment had no effect on this parameter (0.7±0.2 mU/ml, NS *vs* vehicle). The proinflammatory TNF-α content in the graft kidneys was also very low (10.2±1.2 ng/mg protein). Thalidomide treatment did not affect this parameter either (9.7±1.4 ng/mg protein, NS *vs* vehicle). In contrast, thalidomide treatment significantly reduced the VEGF content in the graft kidneys (ng/mg protein: 7.7±1.2 in thalidomide group *vs* 44.0±5.2 in vehicle group, p<0.01; Figure 6C).

**Figure 7:**
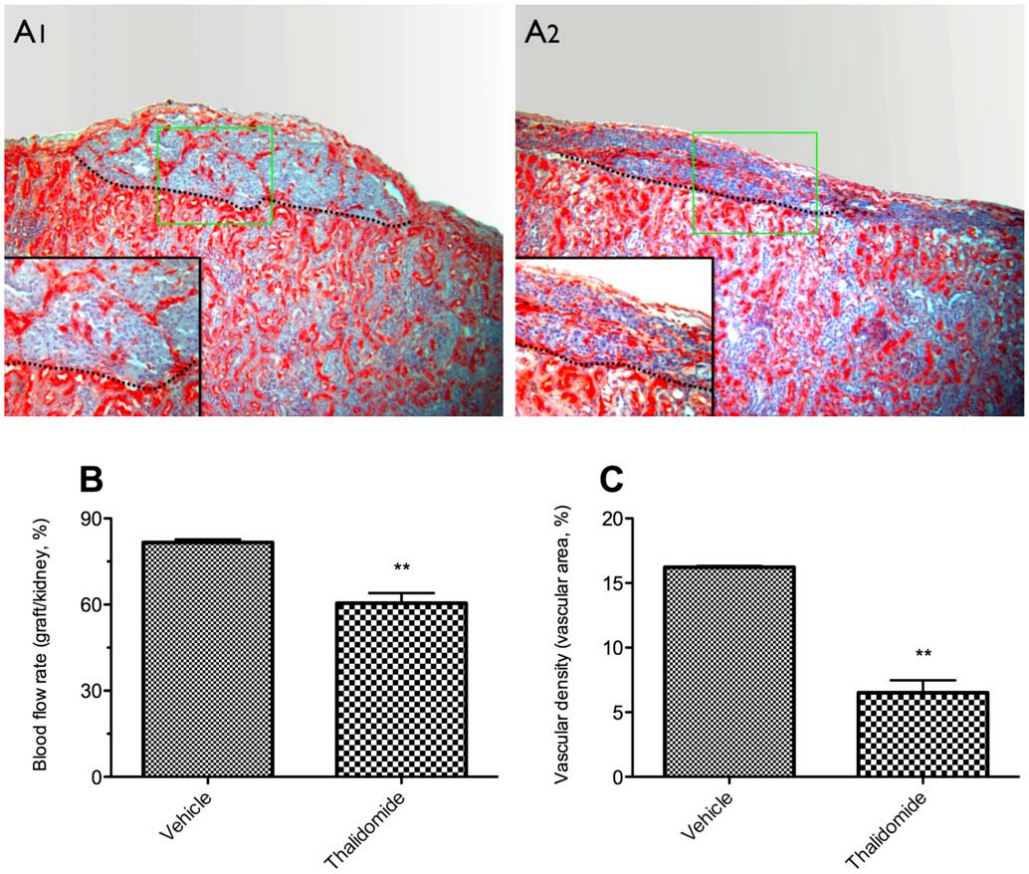
Effects of thalidomide treatment on vascular density and graft surface blood flow in nu/nu mice. Forty-two days post-transplantation, after the blood flow on the graft surface was measured, the grafted kidneys were retrieved and subjected to immunohistochemistry using antibodies against Bandereira simplicifolia (BS-1) lectin. A: Representative pictures of immunostaining of islet graft (dashed line) with BS-1 (red color) at the kidney capsule of nu/nu NMRI mice treated with vehicle (A1) or thalidomide (A2). Magnification ×100 in both pictures, and ×400 in the small pictures inserted. The blood flows were calculated as the percentage of the graft flow rate to kidney surface flow rate (B, n = 6). The vascular density was quantitated as the percentage of lectin-positive area in relation to total section area (C, n = 4). ^**^p<0.01 vs vehicle control graft.

To directly evaluate the effects of thalidomide treatment on islet revascularization, the blood flow on the graft surface was measured before the grafted kidney was removed on day 42. After blood flow measurement, grafted kidneys were retrieved from NMRI nu/nu mice and subjected to immunohistochemical analysis using antibodies against insulin and BS-1 lectin which binds to the luminal surface of endothelial cells [34]. As shown in Figure 7A, thalidomide treatment resulted in an obviously impaired graft vasculature. A lower graft surface blood flow rate was revealed in the thalidomide treated NMRI nu/nu mice group compared to the vehicle group (percentage of graft flow rate to adjacent renal cortex: 60.6±3.5 *vs* 81.4±1.1, p<0.01; Figure 7B). Consistently, a significantly lower vascular density was found in the thalidomide treated nu/nu group versus vehicle animals (percentage of lectin-positive area: 6.5±1.0 *vs* 16.2±0.1, p<0.01; Figure 7C).

## Discussion

This study showed that, together with its inhibitory effect on islet endothelial activation, thalidomide treatment improved islet graft survival and functions in an established xenotransplantation model. In particular, we demonstrated that thalidomide treatment resulted in better blood glucose profile, greater islet graft mass, less leukocyte infiltration and less cytokine content with xenograft in the C57Bl/6 recipient mice. Furthermore thalidomide inhibited the islet graft revascularization as demonstrated in the NMRI nu/nu recipient mice.

In islet transplantation, especially islet xenotransplantation, a cascade of inflammatory reactions occur following transplantation. There is good evidence that activation of the blood coagulation and complement system, and infiltration of the islet graft by neutrophilic granulocytes and macrophages occur immediately after islet transplantation [5], [29]. These infiltrating cells are directly cytotoxic to the islet graft [35]. In addition, these cells produce inflammatory cytokines that lead to apoptosis of islet cells, mainly via TNF-α signaling pathways and upregulation of Fas expression [36], [37]. These pathways induce islet graft apoptosis through activation of nuclear factor-κB (NF-κB)-regulated apoptotic genes and activation of caspases [36], [38]. A third effect of neutrophils and macrophages is their role of antigen-presentation to T-cells. In non-immunosuppressed recipients, islet xenografts reverse diabetes but the majority of transplanted xenogenic islets are subjected to acute cellular rejection mediated by CD4+ and CD8+ T cells and macrophages [39].

Thalidomide treatment protected the islet xenograft in the current study. A rapid graft loss was found in the control C57Bl/6 recipient mice. Shortly after islet transplantation these mice quickly lost the grafts and became diabetic again. Thalidomide-treated mice maintained their functioning islet graft and had much better glucose control. Several anti-inflammatory mechanisms have been investigated here. First, thalidomide treatment dramatically reduced the TNF-α content in the grafted kidneys in the C57Bl/6 mice. TNF-α is a key regulator of other proinflammatory cytokines and of leukocyte adhesion molecules, and is a priming activator of inflammatory cells. Since the TNF–α pathway is one of the major pathways that lead to loss of islet grafts, the inhibitory effect of thalidomide on TNF-α content in islet grafted kidneys in this study may primarily explain the decreased rate of islet graft loss.

Secondly, thalidomide treatment reduced leukocyte infiltration of the islet graft as evidenced by reduced MPO activity. As discussed above, neutrophils are the major effector cells that lead to early graft loss in islet transplantation. The reduced neutrophil infiltration by thalidomide provides additional protection to the islet graft. This is consistent with another study in which thalidomide reduced leukocyte recruitment and tissue damage within the pancreas in a mouse model of acute pancreatitis [24].

Thirdly, thalidomide reduced VEGF content in the grafted kidneys. Although VEGF is essentially required in the islet graft revascularization process, excessive VEGF induces increased microvascular hyper-permeability and activation of the coagulation pathway. In the current study, reduced VEGF content in the graft kidneys by thalidomide treatment is correlated with decreased sluggish blood flow associated with the inflammatory reaction observed in the control C57Bl/6 mice.

These three mechanisms may produce a cumulative protective effect on transplanted tissue, as demonstrated by the facilitation of sustaining islet graft functions in thalidomide-treated C57Bl/6 recipient mice.

In addition to its anti-inflammatory property, thalidomide also possesses an anti-angiogenic potential. The latter was confirmed in both an *ex vivo* islet sprouting model and an *in vivo* islet transplantation model in the current study. In the *ex vivo* islet sprout assay, thalidomide inhibited the sprout formation induced by basic fibroblast growth factor (bFGF) in a dose-dependent manner. TNF-α has been shown to stimulate angiogenesis directly, and also indirectly, by enhancing the synthesis of bFGF [40], [41], [42]. Here, TNF-α increased the sprout formation by 6-fold when used alone and 23-fold when combined with VEGF. The addition of thalidomide to the medium significantly inhibited the increase by 44.2% and 84.8%, respectively. The magnitude of thalidomide's inhibitory effect on VEGF-induced sprout formation was identical to the magnitude of the effect of the Flk-1 inhibitor, SU416, and was reversed by the VEGF-receptor increasing S1P. Similarly, thalidomide's inhibitory effect on TNF-α-induced sprout formation was comparable with the effect of the anti-TNFR1 neutralizing antibody. The combinations of thalidomide with SU416 or anti-TNFR1 did not produce an accumulative inhibitory effect. These results indicate that thalidomide may act through both VEGFR2 and TNFR1 to produce an anti-angiogenesis effect. In the *in vivo* islet transplantation model, thalidomide may have an additive inhibitory effect on endothelial proliferation by reducing the TNF-α/VEGF content in the local graft site and a direct inhibitory effect on TNF-α/VEGF-induced angiogenesis. Although we were unable to compare the revascularization process in the C57Bl/6 mice groups due to the rapid graft loss in the control group, thalidomide demonstrated its anti-angiogenesis effect clearly in the nu/nu mice. The treated mice had greatly impaired islet graft revascularization, evidenced by vascular density count and graft surface blood flow measurement, compared to the control recipients. Since these mice are immunocompromised nude mice and did not mount a cellular immune response of graft rejection, we infer that the impaired islet graft survival in these recipients resulted from the impaired graft revascularization process.

Both anti-inflammatory and anti-angiogenic properties of thalidomide were demonstrated in the current study. It appears that thalidomide can exhibit different characteristics depending on the immune barrier. In the C57Bl/6 mice groups, where rapid graft rejection and inflammatory reactions destroyed the islet graft shortly after transplantation, thalidomide exhibited a mainly anti-inflammatory effect and improved islet graft survival and functions. In the NMRI nu/nu mice, which did not mount a cellular immune response of graft rejection, thalidomide exerted a mainly anti-angiogenic effect.

Interestingly, thalidomide treatment seems to result in adequate blood flow to the islet graft in the immunocompetent C57Bl/6 recipients and protected the graft from the microvascular injury observed in the controls. Those microvessels induced by inflammatory cytokines, such as TNF-α and excessive VEGF at the graft site, were leaky and did not contribute to the required blood supply to the graft [43]. Thalidomide's anti-angiogenic effect may provide an additional protective mechanism in this case by inhibiting islet endothelial activation.

In conclusion, we have demonstrated that *in vivo* administration of thalidomide exerted beneficial effects on the overall results of islet xenotransplantation in diabetic immunocompetent mice. These results suggest that thalidomide could be potentially applied in islet xenotransplantation as an immunomodulator.
