# Correction: Anti-Inflammatory Thalidomide Improves Islet Grafts Survival and Functions in a Xenogenic Environment

**DOI:** 10.1371/annotation/be980555-eeb9-4c0e-91f9-cdc1d8f2709b

**Published:** 2009-11-23

**Authors:** Chunguang Chen, Carina Kuehn, Reinhard G. Bretzel, Thomas Linn

Two funding sources were omitted from the funding statement of this manuscript. The correct funding information statement is: Dr. Linn is supported by a grant of the Faculty of Medicine, Justus Liebig University and the University Medical Center Giessen and Marburg. The funders did not play a role in the study or the preparation of the manuscript.

